# A Framework for the Modulation and Alleviation of Pain Sensations: A Narrative Review

**DOI:** 10.7759/cureus.83508

**Published:** 2025-05-05

**Authors:** Rushita Dobariya, Niraj Kinariwala, Nirav Parekh, Dhruvi Gangani, Devshree Dave, Hasti Maru, Nandani Mangukiya, Siddhi Singh

**Affiliations:** 1 Department of Conservative Dentistry and Endodontics, Karnavati School of Dentistry, Gandhinagar, IND; 2 Department of General Dentistry, Smile Rite Dental Care, Southington, USA

**Keywords:** corticosteroids, cryotherapy, endodontic pain management, low level laser therapy, photobiomodulation

## Abstract

Modern dentistry and endodontic practice is based on effective pain management, which highlights the necessity of a thorough, multifaceted framework to address both acute and chronic pain. In endodontic applications, this narrative review examines pharmacological, non-pharmacological, and integrative approaches for the regulation and reduction of pain sensations by synthesizing data from a variety of investigations. Apart from pharmaceutical treatments like corticosteroids, clonidine-enhanced local anesthetics, and innovative methods that target peripheral and central pain pathways, significant developments include the use of intracanal cryotherapy, low-level laser therapy (LLLT), and photobiomodulation. To shed light on the biological bases of pain, the roles of GABAergic signaling, interleukin-8 regulation, and neurotransmitters such as substance P are investigated in dental pain mechanisms. Additionally, the review highlights the significance of the intensity of pain prior to surgery, the effectiveness of supplemental treatments such as nano-pulsed lasers, and the importance of patient-specific variables in maximizing pain management. This review includes evidence-based, multimodal solutions to improve patient outcomes and promote a pain-free dental experience by combining state-of-the-art clinical trials, systematic reviews, and meta-analyses.

## Introduction and background

One of the main concerns for both patients and dentists is pain, which is a well-acknowledged side effect of orthodontic operations and oral pathological diseases [1]. The experience of dental pain is known to be caused, at least in part, by an inflammatory response involving many molecular processes [2]. Overall, the peripheral pain pathways linked to odontogenic painful disorders are comparable to those found in other areas of the body. The types of sensory neurons involved, and the various molecules that play a role in these processes - such as receptors, channels, transmitters, and intracellular signaling effectors responsible for the transduction, modulation, and propagation of peripheral stimuli - exemplify these similarities [3].

**Figure 1:**
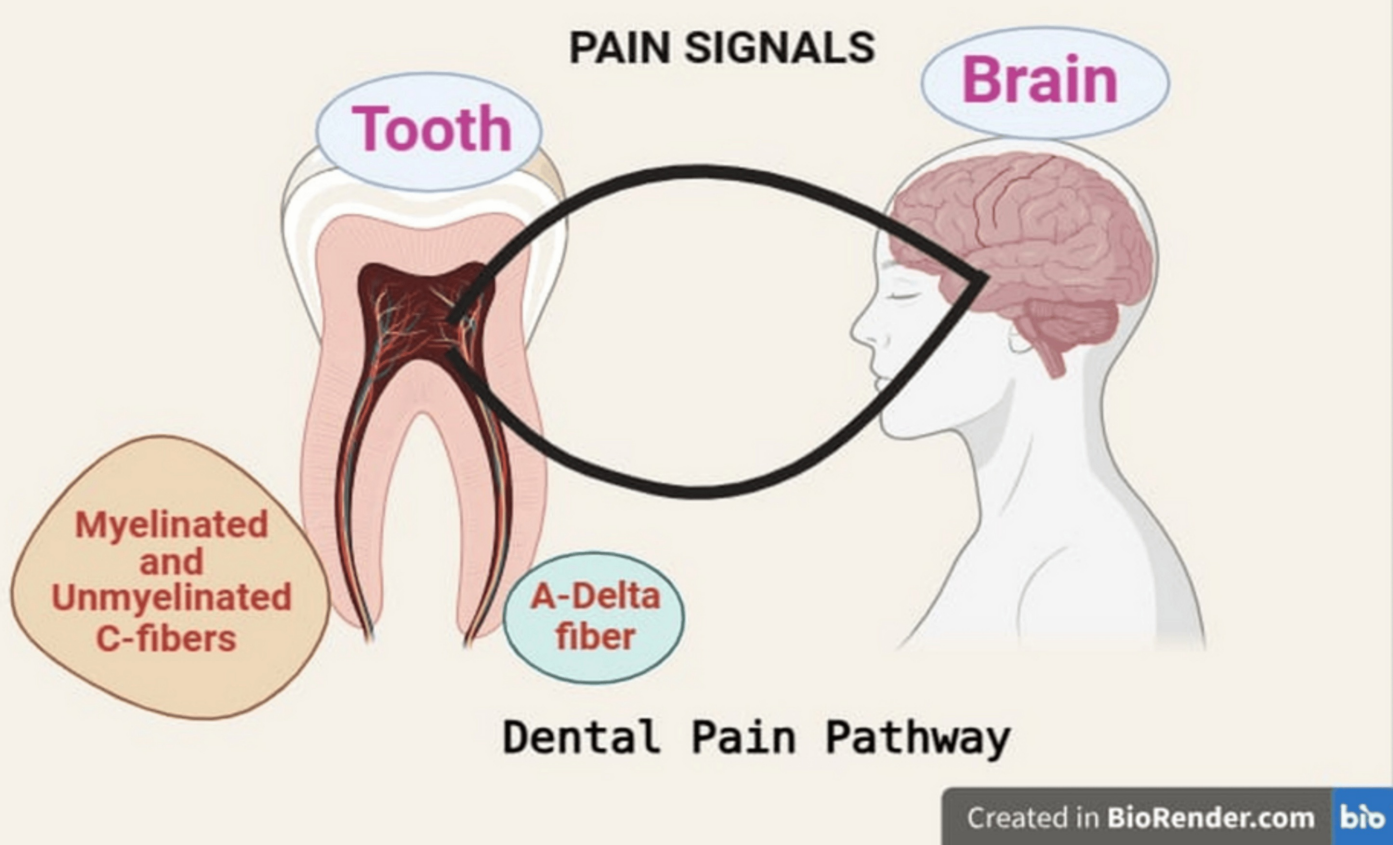
Dental Pain Pathway Image Credit: This figure was generated by Dr. Rushita Dobariya using BioRender (https://www.biorender.com/).

Thin fibers, including both myelinated and unmyelinated C-fibers, carry the pain signal. When local stimuli are detected, an inflammatory reaction is triggered, raising intrapulpal pressure and activating free nerve terminals as a result. Pain can be dull (via C nerve fibers), persistent, sharp (through A nerve fibers), or pulsating, depending on its intensity. Additionally, the multifaceted origin of dental pain - which includes iatrogenic damage, trauma, periodontitis, dental caries (microbial), and other irritants - adds to its complexity (Figure 1) [4].

Pharmacological medications, such as corticosteroids, local anesthetics, and nonsteroidal anti-inflammatory drugs (NSAIDs), have been a major part of traditional pain management strategies [5]. For example, systematic reviews and meta-analyses have shown that corticosteroids are highly effective in reducing postoperative pain and inflammation. Similarly, modifications to local anesthetics, including the combination of clonidine with lidocaine, have been investigated to improve anesthetic efficacy - especially in cases of symptomatic irreversible pulpitis [6].

Non-pharmacological approaches are gaining popularity as supplements or substitutes for traditional therapy, according to new data. For instance, randomized controlled trials have shown that intracanal cryotherapy can effectively reduce postoperative pain by lowering the canal's temperature and reducing inflammatory reactions [7]. Low-level laser therapy (LLLT) is another cutting-edge strategy, as is photobiomodulation, both of which have shown promising results in tissue repair and pain alleviation. Research has also explored the potential of nano-pulsed magneto-infrared laser therapy, which has demonstrated effectiveness in reducing post-endodontic pain when combined with oral analgesics [8].

New targets for pain modulation have also been revealed by developments in molecular research. New routes for intervention include, for example, the role of GABAergic signaling in dental pain and the therapeutic modulation of interleukin-8 (IL-8) pathways [9]. Further insights into the transmission and regulation of pain can be gained by understanding the peripheral processes of odontogenic pain, such as the role of C-fibers and neurotransmitters like substance P [10]. To provide thorough context, this narrative review aims to compile the most recent data from clinical trials, systematic reviews, and molecular investigations.

## Review

Materials and procedures

The purpose of this narrative review was to assess and compile both earlier literature and the most recent data regarding the all-encompassing framework for endodontic pain modulation and alleviation. A comprehensive literature search was conducted using electronic databases - PubMed, Google Scholar, Scopus, and the Cochrane Library - including articles published from 1985 to 2025, with most selected articles being from more recent years. The articles, which included both pharmacological and non-pharmacological methods, were chosen for their applicability to endodontic pain management.

Effective centrally acting and peripherally acting drugs for endodontic pain are essential for the success of dental procedures and the comfort of patients [11]. Pharmaceutical interventions are critical for minimizing the discomfort caused by endodontic procedures, both before and after surgery [12]. This section reviews the main drugs used in endodontic pain control, along with information on their mechanisms, effectiveness, and clinical implications [13].

NSAIDs have analgesic and anti-inflammatory qualities; they are frequently used to treat postoperative endodontic (PE) pain. By blocking the cyclooxygenase enzymes (COX-1 and COX-2), they lower the production of prostaglandins, which are implicated in inflammation and discomfort [14]. One of the most researched NSAIDs for this purpose is ibuprofen. According to Moore et al., NSAIDs, either by themselves or in conjunction with acetaminophen, are safe and effective for managing short-term discomfort during nonsurgical dental treatments [15].

Pharmacological measures

Use of Antibiotics to Treat or Prevent Infections

Antibiotics are used in combination with other analgesics to treat or prevent secondary infections when infection is a contributing factor in pain. Antibiotics can help avoid problems that could make pain worse, even though they are not used directly to treat pain (e.g., metronidazole, amoxicillin, and clindamycin) [16]. These are recommended for patients with acute or chronic apical periodontitis or any surgical infection symptoms. One important consideration is that antibiotics are usually only used when an infection is clearly present, because overuse might result in resistance and gastrointestinal adverse effects [17].

Paracetamol or Acetaminophen

Another analgesic that is frequently used in conjunction with NSAIDs to improve pain relief is acetaminophen. It is thought to inhibit central prostaglandin synthesis, though its precise mechanism is yet unclear. It has been demonstrated that acetaminophen and NSAIDs work better together to manage pain than either medication does by itself [18].

Corticosteroid

Strong anti-inflammatory drugs called corticosteroids can be used locally or systemically to treat endodontic discomfort [19]. They work by blocking phospholipase A2, which lowers the synthesis of inflammatory mediators. The pharmacology and mechanisms of corticosteroids are also indicated for endodontics [20]. 

Diclofenac Sodium

This particular NSAID is frequently used to treat postoperative dental pain and swelling because it works by inhibiting the Cox enzymes [21]. By drastically lowering a patient's opioid needs, postoperative NSAIDs, such as diclofenac sodium, can lessen the frequency and intensity of opioid-induced adverse events (AEs) [22]. One study examined how well diclofenac sodium worked to treat pain and contrasted the effects of oral and intraligamentary administration. According to the study, diclofenac sodium administered prophylactically intraligamentarily reduced PE more effectively than oral treatment [23].

Opioid Painkillers

Strong analgesics called opioids are frequently used to treat moderate to severe pain, especially when acetaminophen and NSAIDs are not enough [24]. They block pain signals by acting on the central nervous system. Codeine, hydrocodone, and morphine are a few examples [25]. Indications, such as when endodontic treatment results in severe postoperative pain, mean they are usually administered for a brief period of time. One important consideration is that opioids should be used carefully and usually in combination with other analgesics to lower their necessary dose, due to their potential for addiction and adverse effects, such as drowsiness and constipation [26].

Gabapentin and Pregabalin Are Gabapentinoids

When nerve damage is a concern, gabapentinoids, which are anticonvulsants, can be used to treat neuropathic pain that may follow endodontic operations. Inhibiting excitatory neurotransmitters in the brain and spinal cord is how these medications function (pregabalin and gabapentin) [27]. Indications are that they are frequently used to treat individuals who have chronic pain after receiving endodontic treatment or to manage pain related to nerve injury. Though these medications are well tolerated, they should be closely monitored for adverse effects, such as dizziness, somnolence, and other effects on the central nervous system [28].

Relaxants for the Muscles

Endodontic patients who exhibit muscle spasms or discomfort may occasionally be treated with muscle relaxants, especially if they have been in a position for a long time during treatment [29] (for instance, medications such as cyclobenzaprine and diazepam). Indications are that they are used to relax muscles in temporomandibular joint issues or myofascial discomfort. Considering the possibility of adverse effects, including fatigue and lightheadedness, they are typically used in the short term [30]. 

Anticonvulsants for Neuropathic Pain

Anticonvulsants like carbamazepine and phenytoin are used in patients with persistent or severe neuropathic pain after endodontic treatments, particularly in those who may have nerve damage or post-surgical complications (e.g., carbamazepine and phenytoin) [30]. Indications are that they are effective in managing neuralgic pain, including post-treatment neuralgia or conditions like trigeminal neuralgia. One important consideration is that long-term use can have significant side effects, such as dizziness, ataxia, and blood dyscrasias [31].

Analgesics With Central Action - Tramadol

Tramadol is a centrally acting analgesic that works by blocking the reuptake of serotonin and norepinephrine, as well as by acting on opioid receptors [32]. It is frequently used to treat moderate pain when acetaminophen and NSAIDs are not enough. Despite being less addictive than conventional opioids, tramadol can nevertheless have negative side effects, including nausea, lightheadedness, and an increased risk of seizures [33]. 

Antihistamines

Antihistamines are mainly used to treat allergic responses, but they are also occasionally used in endodontics to treat postoperative pain, particularly in patients who have previously experienced adverse reactions to other painkillers [34]. They also aid in reducing related symptoms, including inflammation and edema, and examples include hydroxyzine and diphenhydramine. They are useful for treating pain from allergic responses or for reducing inflammation when combined with other painkillers. A common side effect of antihistamines is sedation, which may not be suitable for every patient [35].

Topical Anesthetics

To lessen discomfort related to postoperative sensitivity, topical anesthetics can be administered to the mucous membranes or dentin surface, in addition to the conventional local anesthetics used during treatment (e.g., lidocaine gel and benzocaine) [36]. These can be administered topically to offer temporary alleviation [37].

Local Anesthesia

In order to control pain during endodontic treatments, local anesthetics are necessary. They stop the propagation of nerve impulses by blocking sodium channels. Lidocaine is frequently used in conjunction with epinephrine to extend the effects of anesthesia and lessen discomfort during surgery [38]. Anatomical variances and inflammation are two examples of factors that can affect how effective local anesthetics are. Three combination treatments improved pain outcomes significantly by enhancing analgesic effectiveness through synergistic mechanisms [39]. Improved pain control can result from the combination of various analgesic classes. For example, it has been demonstrated that NSAIDs and acetaminophen increase the effectiveness of analgesia. Furthermore, analgesics administered prior to surgery can aid in lessening the severity of postoperative pain [40]. 

Observations and Suggestions

Clinicians should take patient-specific aspects, including medical history, allergies, and possible drug interactions, into account when choosing pharmacological drugs for endodontic pain management [41]. Following dose recommendations and evidence-based guidelines is crucial to maximizing effectiveness and minimizing side effects. Several different drugs are utilized for preoperative and postoperative pain treatment in endodontics, in addition to widely used ones like NSAIDs, acetaminophen, corticosteroids, and local anesthetics [42]. Depending on how they work and the type of pain they treat, these drugs can be divided into a number of groups. The following are some of the main medications used to treat endodontic pain [43].

Clinical management of pain following root canal therapy

An essential part of endodontic care is pain management after root canal therapy, which aims to improve patient comfort and encourage healing [44]. A multimodal strategy that incorporates both pharmaceutical and non-pharmacological tactics is necessary for effective therapeutic therapy. The main clinical management techniques based on the most recent data are described in this section [45].

Management of preoperative pain

Preemptive analgesia, such as blocking the pain pathway early on, involves administering analgesics prior to the procedure to help minimize postoperative discomfort. Preoperative NSAIDs, such as 400-600 mg of ibuprofen, are frequently used to manage inflammation and hyperalgesia [46]. Local anesthetic techniques can also provide extended postoperative pain relief; for example, preoperative infiltration with long-acting anesthetics, such as bupivacaine, is often used [47].

Management of intraoperative pain

In endodontics, guaranteeing patient comfort and enhancing treatment outcomes depend on efficient intraoperative pain control. For teeth that are particularly difficult to anesthetize, methods such as additional intraligamentary or intraosseous injections can be used to provide profound anesthesia, which is an essential first step [48]. Additionally, a major factor in reducing pain during treatment is the use of intracanal medications. One popular intracanal medication that aids in pain management is calcium hydroxide, which lowers inflammation and the bacterial load [49]. Corticosteroid-containing pastes are also useful for reducing inflammation and easing postoperative pain [50]. Furthermore, the use of cold saline irrigation during instrumentation (intracanal cryotherapy) has been shown to dramatically reduce postoperative discomfort by lowering inflammation and tissue damage. When combined, these techniques offer a comprehensive approach to controlling discomfort during endodontic procedures [51].

Management of postoperative pain

Non-Pharmacological Measures: LLLT

Endodontic pain can be effectively managed using LLLT, also known as photobiomodulation therapy [52]. This non-invasive technique uses low-energy laser light to alter cellular activity. LLLT works primarily through photochemical interactions, whereby cellular chromophores, such as mitochondrial cytochrome c oxidase, absorb light and subsequently generate additional ATP [53]. This lessens pain and inflammation by improving cellular regeneration and repair, and by lowering inflammatory mediators like prostaglandins and cytokines. Furthermore, by preventing the transmission of pain and promoting the release of endorphins, LLLT alters brain activity. Clinically, LLLT can be used to minimize pain and swelling after surgery, reduce inflammation before surgery, and reduce bacterial load and enhance periapical tissue repair during surgery [54].

Additionally, it has demonstrated promising improvement in tissue healing in retreatment patients and in decreasing the frequency of flare-ups. Because LLLT encourages angiogenesis and cell proliferation, patients frequently report significantly less pain and faster healing compared to traditional techniques [55]. The therapy is a favored supplementary treatment in endodontics, since it is non-invasive and can reduce the need for pharmaceutical interventions. Widespread adoption is nevertheless hampered by issues such as expensive equipment, inconsistent practices, and the requirement for operator training [56].

All things considered, LLLT is a promising development in the treatment of endodontic pain. Its advantages, such as reduced postoperative pain and faster recovery, highlight its potential as an adjunct to traditional mechanical and pharmaceutical approaches. Additional investigation and standardization of protocols are essential to maximize its clinical efficacy and accessibility [57]. It may be possible to target affected tissues more precisely by combining LLLT with advanced imaging methods, such as cone-beam computed tomography (CBCT). Its potential for improved outcomes when combined with nanotechnology and regenerative endodontics is also being explored in ongoing research [58].

Cryotherapy

Both intraoral and extraoral cryotherapy effectively promote analgesia and reduce inflammation. In endodontics, cryotherapy - the use of low temperatures - has drawn interest as a complementary method for treating post-treatment discomfort [59]. During root canal therapy, this method uses cold irrigation solutions, such as cryotreated sodium hypochlorite or cold saline. Cryotherapy's main mechanism is its capacity to lower tissue temperature, which causes vasoconstriction, a decrease in metabolic activity, and a reduction in the release of inflammatory mediators. Together, these actions reduce nerve conduction velocity, tissue edema, and pain perception [60].

After biomechanical preparation, the root canal system is usually irrigated with cold saline at temperatures between 2°C and 4 °C to perform intracanal cryotherapy in clinical practice. Research has indicated that this technique significantly lowers postoperative pain compared to the use of room-temperature irrigation solutions. Furthermore, it has been noted that cryotherapy enhances patient comfort during the crucial 24 to 48 hours following treatment, which is frequently associated with increased pain and inflammation [61].

The benefits of cryotherapy go beyond just pain relief; by lowering inflammation in the periapical tissue, it also speeds up healing. Additionally, because it is a non-pharmacological modality, it is a desirable choice for individuals who are contraindicated for some analgesics or who prefer to use minimal amounts of medicine [62]. Notwithstanding its benefits, the method needs standardization of procedures, such as ideal chilling temperatures and times, in order to produce reliable results. All things considered, cryotherapy is an easy, economical, and effective way to improve postoperative outcomes in endodontic treatment [63]. Its capacity to lessen discomfort and reduce the intensity of postoperative pain further highlights its potential as a useful adjuvant in contemporary endodontic practice [64].

Reducing the possibility of pain following endodontic treatment requires the use of preventive measures. The control of occlusal interference is a crucial first step [65]. Adjusting the occlusion to remove early contacts ensures a more comfortable recovery for the patient, helping to avoid further discomfort from mechanical trauma to the treated tooth. Achieving and preserving an appropriate coronal seal is another crucial preventive step. By doing so, the root canal system is shielded against bacterial reinfection, which frequently results in postoperative discomfort and treatment failure. Clinicians can improve treatment outcomes and reduce patient discomfort by addressing these factors [66].

Patient Education and Monitoring

A key element of effective endodontic pain management is patient education and appropriate follow-up; clear instructions for at-home care should be given to patients. These instructions should cover things like how to take prescription drugs as directed, how to avoid hard or sticky meals, and how to maintain good oral hygiene [67]. In order to avoid putting unnecessary strain on the healing tissues, it is especially crucial to encourage patients to limit excessive chewing on the treated tooth. Furthermore, planned follow-up visits are essential for tracking the patient's development. In order to guarantee the best possible healing and patient satisfaction, these visits enable clinicians to see possible issues early on, such as flare-ups or chronic pain, and to take prompt action [68].

Management of persistent pain

If pain doesn't go away after treatment, more procedures may be needed to address the underlying problems. Repeating endodontic operations is one strategy, especially if the discomfort is caused by insufficient root canal system cleaning, shaping, or sealing. Retreatment makes it possible to remove any leftover debris, bacteria, or filling materials that might be causing the pain. Surgical procedures, like apicoectomy or peri-radicular surgery, may be considered if conservative methods, such as retreatment, are ineffective in reducing the discomfort. These techniques provide direct access to treat periapical pathology by focusing on chronic infection or inflammation near the root apex [69].

Furthermore, if non-endodontic causes of the pain are suspected, such as temporomandibular disorders or neuropathic pain, a referral to a specialist is frequently required. In order to treat complicated or overlapping disorders that could mimic or worsen endodontic pain, this ensures that the patient receives the proper multidisciplinary care. Clinicians can effectively manage chronic pain and enhance patient outcomes by using a thorough and focused strategy [70].

## Conclusions

Pharmacological, non-pharmacological, and preventive measures must all be integrated into a thorough and multidimensional approach for endodontic pain management to be effective. From preoperative planning to postoperative care, this review emphasizes how critical it is to handle pain at every stage of endodontic treatment. Both intraoperative and postoperative pain have been shown to be significantly reduced by methods such as LLLT, cryotherapy, profound anesthesia, and intracanal drugs. By reducing issues, preventive procedures, like occlusal adjustments, and ensuring coronal seals are in place, improve patient outcomes even more. Furthermore, adherence to recommended regimens and early detection of possible problems are ensured by patient education and routine follow-ups.

New approaches to pain management, like photobiomodulation and sophisticated pharmaceuticals, present encouraging opportunities. But more investigation and established procedures are necessary to maximize various methods and guarantee their broad clinical relevance. Clinicians can successfully reduce pain, increase treatment success, and enhance the overall patient experience in endodontic care by implementing an evidence-based and patient-centered strategy.
